# Effectors of Hyperlipidemia Among Patients with HIV/AIDS Taking Second-line Antiretroviral Therapy Based on Registry Data

**DOI:** 10.2174/1570162X20666220805103411

**Published:** 2022-11-30

**Authors:** Chunling Yang, Dongli Wang, Yanmin Ma, Zhibin Liu, Huijun Guo, Feng Sang, Qianlei Xu, Yantao Jin

**Affiliations:** 1 The First Clinical Medical School, Henan University of Chinese Medicine, Zhengzhou, China;; 2 Center for AIDS/STD Control and Prevention, Center for Disease Control and Prevention of Henan Province, Zhengzhou, China;; 3 Department of Acquired Immune Deficiency Syndrome Treatment and Research Center, The First Affiliated Hospital of Henan University of Chinese Medicine, Zhengzhou, China;; 4 Henan Key Laboratory of Viral Diseases Prevention and Treatment of Traditional Chinese Medicine, Henan University of Chinese Medicine, Zhengzhou, China

**Keywords:** HIV, AIDS, second-line ART, hyperlipidemia, cross-sectional study, mortality

## Abstract

**Objective:**

In this study, we aimed to determine the prevalence and effectors of hyperlipidemia among People Living with HIV/AIDS (PLWHA) and taking second-line Antiretroviral Therapy (ART) using registry data in central China.

**Methods:**

We conducted a cross-sectional study and collected information of PLWHA on second-line ART during 2018 from two medical registries. Hyperlipidemia was defined according to the 2016 Chinese guidelines for the management of dyslipidemia in adults. Univariate and multivariate logistic regression analyses were performed to explore the influencing factors of hyperlipidemia. We calculated Odds Ratios (ORs) and 95% Confidence Intervals (CIs).

**Results:**

A total of 2886 PLWHA taking second-line ART were included in this study, and 978 (33.9%) had hyperlipidemia. Female patients, those with hyperglycemia, and patients with CD^4+^ cell counts >500 cells/μL had a higher prevalence of hyperlipidemia with 37.0%, 49.0%, and 41.3%, respectively. Multivariate analysis showed that CD^4+^ cell count 350-500 cells/μL (OR = 1.72, 95% CI: 1.26-2.38), CD^4+^ cell count >500 cells/μL (OR = 2.49, 95% CI: 1.85-3.38), and FPG >6.2 mmol/L (OR = 2.08, 95% CI:1.64-2.65) were risk factors for hyperlipidemia. Male sex (OR = 0.72, 95% CI: 0.61-0.85) and Hb <110 g/L (OR = 0.59, 95% CI: 0.45-0.76) were protective factors against hyperlipidemia.

**Conclusion:**

PLWHA on second-line ART had a higher prevalence of hyperlipidemia. Gender, CD^4+^ cell count, FPG, and hemoglobin were influencing factors of hyperlipidemia.

## 1 INTRODUCTION

Acquired immune deficiency syndrome (AIDS), caused by the human immunodeficiency virus (HIV), is a major public health threat, with an estimated 38 million individuals affected as of the end of 2019 [1]. The introduction and scale-up of antiretroviral therapy (ART) have substantially reduced AIDS-related mortality and mortality among people living with HIV/AIDS (PLWHA) [2], transforming AIDS into a chronic disease [3]. As life expectancy is increasing overall, the emergence of non-AIDS-related clinical events has become the main factor affecting mortality and survival in PLWHA [4, 5]. Cardiovascular disease (CVD) has become the leading cause of death among PLWHA in recent years [2, 6], and the major driving factor is hyperlipidemia [7]. Hyperlipidemia refers to elevated triglycerides (TG), elevated total cholesterol (TC), or both [8]. It is common among PLWHA who are taking ART, especially protease inhibitor-based ART [9, 10]. With an increased duration of first-line ART, non-adherence to treatment, drug resistance, and failed therapy become more common, leading to PLWHA changing to second-line ART, as recommended by the World Health Organization (WHO) [11]. Thus, greater attention is needed regarding the status of blood lipids associated with second-line ART. Studies have revealed that among PLWHA taking second-line ART, the overall prevalence rate of hyperlipidemia is between 28% and 80% [12], occurring as early as 4 weeks after beginning the ART regimen [13]. There is not much data on the prevalence of hyperlipidemia among PLWHA taking second-line ART in China; thus, the influencing factors of hyperlipidemia in this population have not been fully identified.

Henan Province is an HIV epidemic area in China owing to illegal blood plasma collection during the 1990s [14]. Henan is also one of the first regions in China to promote free ART for PLWHA. Since 2009, more PLWHA with first-line ART failure in this region have gradually switched to second-line ART. Thus, we conducted a cross-sectional study in this region to determine the prevalence and influencing factors of hyperlipidemia among PLWHA on second-line ART, with the aim to help reduce the risk of CVD and prolong the lifespan of PLWHA through early prevention and treatment of hyperlipidemia.

## 2 MATERIALS AND METHODS

### 2.1 Study Setting

This study was conducted in Henan Province, in central China, where most PLWHA were infected with HIV through illegal commercial plasma/blood collection during the 1990s [14]. The Chinese government established the National Free Antiretroviral Treatment Program (NFATP) in 2003 to provide free ART for all PLWHA. With the failure of first-line ART, some PLWHA switched to second-line ART, beginning in 2009. Before changing to second-line therapy, patients’ first-line ART comprised zidovudine/lamivudine (3TC)/tenofovir *disoproxil fumarate* (TDF)/stavudine/ abacavir/ didanosine in combination with nevirapine or efavirenz. After switching, all patients’ second-line ART comprised 3TC + TDF + lopinavir/ritonavir (Lpv/r). Additionally, the National Chinese Medicine Treatment AIDS Trial Program (NCMTATP) was initiated as an alternative therapy for PLWHA. PLWHA could voluntarily participate in the NCMTATP, which involved taking *Yi Ai Kang* capsules, a Chinese patent drug specifically used for treating HIV infection, at no cost to the patient until they left the NTCMTP. Henan Province was one of the earliest regions to participate in the program [15, 16]. In our study, we used the standard medical records registered in the NFATP and NCMTATP reported elsewhere [16].

### 2.2 Study Population

We conducted a retrospective cross-sectional study using the information on PLWHA collected from the standard medical records of the NFATP and NCMTATP in 2018. All individuals in this study were diagnosed with HIV using western blot, received second-line ART before June 2018, were aged between 15 and 60 years, and had records for TG and TC during 2018. PLWHA missing the variables of interest were excluded from the study.

The sample size was calculated according to the following formula:


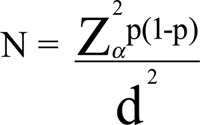


where α = 0.05, d = 0.1, and *p* = 0.27 [17]. The minimum sample size was calculated to be 1039 patients needed to explore the effectors of hyperlipidemia among PLWHA. As a real-world study using standard medical registries, the sample in this study should be more than 1039 participants.

### 2.3 Data Collection and Variables

We collected information of PLWHA including date of birth, gender, marital status, ethnicity, education, occupation, route of infection, date HIV-positive status was confirmed, dates first-line ART and second-line ART were started, whether taking Chinese medicine (CM), CD^4+^ cell count, hemoglobin (Hb), fasting plasma glucose (FPG), TG, and TC from the registries of the NCMTP and NCMTATP. We used values for CD^4+^ cell count, Hb, FPG, TG, and TC recorded within 6 months from 1 June 2018. If no values for any variable were entered during this interval, the variable was defined as missing. The criteria for hyperlipidemia were defined as TC >6.20 mmol/ L and/or TG >2.30 mmol/L, according to the 2016 Chinese guidelines for the management of dyslipidemia in adults.

### 2.4 Data Analysis

Qualitative data are described as frequency and percentage. Differences in sociodemographic, clinical, and immunological characteristics between patients with hyperlipidemia and those without hyperlipidemia were calculated and compared using the chi-square test. Logistic regression in univariate and multivariate analyses was used to explore the influencing factors of hyperlipidemia among PLWHA taking second-line ART. In the logistic regression models, hyperlipidemia was used as the dependent variable and the remaining variables as covariables. The results are shown as odds ratio (OR) and 95% confidence interval (CI). The data were analyzed using R 3.6.1 software (The R Project for Statistical Computing, Vienna, Austria). We considered *P*<0.05 to indicate statistical significance.

## 3 RESULTS

### 3.1 Summary of Study Population

A total of 2886 PLWHA met our inclusion criteria and were enrolled in this study. Of those, 978 had hyperlipidemia (Hyperlipidemia group) and 1908 (Non-hyperlipidemia group) did not. The prevalence of hyperlipidemia was 33.9%. The prevalence of hypertriglyceridemia combined with hypercholesterolemia, isolated hypertriglyceridemia, and isolated hypercholesterolemia was 5.1%, 31,5%, and 7.3%, respectively.

The average age of participants was 51.4 ± 7.7 years, with most between the ages of 45 and 55 years. In the Non-hyperlipidemia group, 1368 (71.7%) PLWHA had completed more than 6 years of education; this number was 727 (74.3%) in the Hyperlipidemia group. Most (83.4%) PLWHA were married. A total of 2842 (98.5%) PLWHA had been diagnosed with AIDS for more than 5 years, 699 (24.2%) had received ART for more than 8 years before starting second-line ART, 2575 (89.2%) had received second-line ART for more than 3 years, and 547 (19.0%) had received CM therapy. In total, 325 (11.3%) participants had FPG >6.2 mmol/L and 381 (13.2%) had Hb ≤110 g/L. The number of PLWHA with baseline CD^4+^ T-cell counts <200, 200-350, 351-500, and >500 cells/μL was 314, 572, 712, and 1288, respectively. Details regarding the general characteristics of PLWHA taking second-line ART in our study are summarized in Table **1**.

### 3.2 Influencing Factors of Hyperlipidemia Among PLWHA on Second-line ART

Our results showed that female patients, those with hyperglycemia, and patients with baseline CD^4+^ T-cell count >500 cells/μL had a higher prevalence of hyperlipidemia, with rates of 37.0%, 49.0%, and 41.3%, respectively. The results of univariate logistic regression analysis showed that gender, CM therapy, CD^4+^ cell count, FPG, and Hb were risk factors for hyperlipidemia in PLWHA (*P*<0.05). The risk of hyperlipidemia was 0.73 greater in male than in female patients, 2.04 times higher in patients with FPG >6.2 mmol/L, and 0.58 times greater in patients with Hb ≤110 g/L. Additionally, the risk of hyperlipidemia in patients with baseline CD^4+^ T-cell count >500 cells/μL was 2.69 times higher than that in patients with baseline CD^4+^ T-cell counts <200 cells/μL. After adjusting for other confounding factors in multivariable logistic regression, gender, CD^4+^ cell count, FPG, and Hb were found to be independent influencing factors of hyperlipidemia (*P*<0.05). Female patients with baseline CD^4+^ T-cell count >500 cells/μL, those with FPG >6.2 mmol/L, and patients with Hb ≤110 g/L had 0.72 times, 2.49 times, 2.08 times, and 0.59 times higher risk of developing hyperlipidemia, respectively. The detailed results of the logistic analysis are shown in Table **2**.

## 4 DISCUSSION

The WHO has proposed a public health approach to promote ART therapy in developing countries. The recommended first-line ART consists of two nucleoside or nucleotide reverse transcriptase inhibitors (NRTIs) plus a non-NRTI. If first-line ART fails, the WHO recommends changing to a ritonavir-boosted protease inhibitor plus two NRTIs as second-line ART [18, 19]. Dyslipidemia is more common in PLWHA taking protease inhibitor-based second-line ART. In our study, we aimed to determine the prevalence and influencing factors of hyperlipidemia among PLWHA taking second-line ART in central China to provide a reference for early prevention and treatment of hyperlipidemia in clinical practice.

Our study showed that the prevalence of hyperlipidemia in PLWHA taking second-line ART was 33.9%. A study that included 1537 participants with sexually transmitted HIV/AIDS in the same area as our study showed that the prevalence of hyperlipidemia in PLWHA taking first-line ART was 19% and that in PLWHA taking second-line ART was 30.4% [20]. Other studies have reported a prevalence of hyperlipidemia among PLWHA on Lpv/r-based ART of 43.9% in Tanzania [21], 65% in Spain [22], 82% in Italy [23], and 29% in South Africa [24]. A retrospective study showed that the prevalence of hyperlipidemia among PLWHA taking Lpv/r monotherapy ranged from 31%-80% [25]. Regardless of whether participants in the above studies were taking Lpv/r alone or in combination, most studies demonstrated a higher prevalence of hyperlipidemia than our findings. Previous studies have demonstrated that the combined use of TDF is associated with a more favorable lipid profile [26]. All patients in our study had TDF included in their treatment regimen, which may be a reason for our findings; this must be further confirmed in future research. The varied prevalence of hyperlipidemia in our study was consistent with the results of previous studies reporting a hyperlipidemia prevalence ranging from 28% to 80%. This difference could be partly explained by the different combinations of antiretrovirals, lifestyles, and differing genetic susceptibility [27] but is mainly owing to different definitions and standards of hyperlipidemia as well as different study designs [25].

In our study, the prevalence of hypertriglyceridemia combined with hypercholesterolemia, isolated hypertriglyceridemia, and isolated hypercholesterolemia was 5.1%, 31.5%, and 7.3%, respectively. The results of a national survey in China in 2012 showed that the overall incidence of hypercholesterolemia and hypertriglyceridemia was 4.90% and 13.1%, respectively [28], which were lower than the prevalence among PLWHA in this study. This suggests a positive correlation between second-line ART and lipid disorders. A prospective study including 212 PLWHA taking PI-based ART showed that the overall incidence of hypercholesterolemia, hypertriglyceridemia, mixed hypercholesterolemia, and hypertriglyceridemia was 38.2%, 25%, and 15.1%, respectively [29]. Another prospective study found that in PLWHA taking Lpv/r-based ART, the prevalence of hypertriglyceridemia was 40% and that of hypercholesterolemia was 17.4% [22]. Although the incidence of hypertriglyceridemia and hypercholesterolemia are inconsistent among different studies, all studies show that the rates of hypertriglyceridemia incidence are very high with PI-based ART. In contrast, hypercholesterolemia was relatively low in our study, which was consistent with recent studies showing that PI-based ART could raise levels of cholesterol and triglycerides in PLWHA and is most closely related to the increase in hypertriglyceridemia [30].

We also found that gender, CM therapy, CD^4+^ T-cell count, FPG, and Hb were risk factors for the prevalence of hyperlipidemia in PLWHA (*P*<0.05). Female patients, those with baseline CD^4+^ T-cell count >500 cells/μL, patients with FPG >6.2 mmol/L, and those with Hb ≤110 g/L had 0.72 times, 2.49 times, 2.08 times, and 0.59 times higher risk of developing hyperlipidemia, respectively. These results were consistent with those of previous studies reporting that female PLWHA with high CD^4+^ T-cell counts, anemia, and hyperglycemia are more likely to have dyslipidemia [21, 31, 32].

CM has an important role in the treatment of HIV/AIDS in China, showing a good curative effect in ameliorating symptoms and signs, improving quality of life, increasing CD^4+^ T-cell counts, and prolonging survival in PLWHA [16]. However, we did not find a relationship between CM and hyperlipidemia after adjusting for other confounding factors in multivariable logistic regression. Previous studies have also shown that age, being married, and low education levels are statistically associated with dyslipidemia [21, 33], but we did not reach such a conclusion.

As observational studies and randomized trials have shown, statins and fibrates have significant effects on reducing levels of TC and TG and are recommended as a cornerstone therapy for dyslipidemia [34]. Nevertheless, the potential pharmacological interactions between ART and PIs [35], as well as those between fibrates and statins [36], are harmful and cannot be ignored. In the early stages, a low-fat diet and increased physical exercise are enough to control the further development of dyslipidemia. Therefore, early intervention is important. We suggest strengthening the detection of blood lipid levels in PLWHA by taking second-line ART.

As a retrospective cross-sectional study based on standard medical record registries, some limitations could not be avoided in this study. The population was a convenience sample and excluded patients with missing information, which could lead to selection bias. Because the data were based on routine medical records, information such as body mass index, smoking, drinking, and dietary factors associated with hyperlipidemia were not assessed in this study.

## CONCLUSION

In our study, PLWHA taking second-line ART had a higher prevalence of hyperlipidemia. Gender, CD^4+^ cell count, FPG, and Hb were influencing factors of hyperlipidemia. However, owing to the limitations in this cross-sectional study based on standard medical record registries, the results of this study need to be confirmed in cohort studies to detect a change in the incidence of hyperlipidemia with second-line ART.

## Figures and Tables

**Table 1:** General characteristics of PLWHA on second-line ART.

**Project**	**Total (2886)**	**Non-hyperlipidemia (1908)**	**Hyperlipidemia (978)**	***χ* ^2^**	***P***
**Gender**					
Female	1590 (55.1%)	1001 (52.5%)	589 (60.2%)	0.729	<0.001
Male	1296 (44.9%)	907 (47.5%)	389 (39.8%)		
**Age (year)**					
<45	496 (17.2%)	322 (16.9%)	174 (17.8%)	0.400	0.819
45-55	1565 (54.2%)	1037 (54.4%)	528 (54.0%)		
>55	825 (28.6%)	549 (28.7%)	276 (28.2%)		
**Educational Status**					
≤6years	791 (27.4%)	540 (28.3%)	251 (25.7%)	2.260	0.133
>6years	2095 (72.6%)	1368 (71.7%)	727 (74.3%)		
**Marital Condition**					
Married	2408 (83.4%)	1591 (83.4%)	817 (83.5%)	0.011	0.917
Sig/window	478 (16.6%)	317 (16.6%)	161 (16.5%)		
**HIV Positive Time (Year)**					
<5	44 (1.5%)	33 (1.7%)	11 (1.1%)	2.609	0.271
5-10	2494 (86.4%)	1637 (85.8%)	857 (87.6%)		
>10	348 (12.1%)	238 (12.5%)	110 (11.3%)		
**ART Time before Second-line (Year)**					
<5	1002 (34.7%)	670 (35.1%)	332 (33.9%)	1.691	0.429
5-8	1185 (41.1%)	790 (41.4%)	395 (40.4%)		
>8	699 (24.2%)	448 (23.5%)	251 (25.7%)		
**Second-line ART Time (Year)**					
<3	311 (10.8%)	204 (10.7%)	107 (10.9%)	0.060	0.970
3-5	1503 (52.1%)	993 (52.0%)	510 (52.1%)		
>5	1072 (37.1%)	711 (37.3%)	361 (37.0%)		
**CM Therapy**					
No	2339 (81.0%)	1568 (82.2%)	771 (78.8%)	1.238	0.030
Yes	547 (19.0%)	340 (17.8%)	207 (21.2%)		
**CD4 Cell Count (cells/μL)**					
<200	314 (10.9%)	249 (13.1%)	65 (6.6%)	70.600	<0.001
200-350	572 (19.8%)	420 (22.0%)	152 (15.5%)		
351-500	712 (24.7%)	483 (25.3%)	229 (23.4%)		
>500	1288 (44.6%)	756 (39.6%)	532 (54.5%)		
**FPG (mmol/L)**					
≤6.2	2561 (88.7%)	1742 (91.3%)	819 (83.7%)	36.955	<0.001
>6.2	325 (11.3%)	166 (8.7%)	159 (16.3%)		
**Hemoglobin (g/L)**					
>110	2505 (86.8%)	1619 (84.9%)	886 (90.6%)	18.590	<0.001
≤110	381 (13.2%)	289 (15.1%)	92 (9.4%)		

**Note:** Values in the table are n (%).

**Abbreviations:** CM, Chinese Medicine ART, Antiretroviral Therapy, HIV, Human Immunodeficiency Virus, FPG, Fasting Plasma Glucose, PLWHA, People Living with HIV/AIDS.

**Table 2:** Binary logistic regression analysis of the influencing factors of hyperlipidemia.

**Project**	**Prevalence**	**Univariate Analysis**		**Multivariate Analysis**	
	**(%)**	**OR(95%CI)**	***P*-Value**	**OR(95%CI)**	***P*-Value**
**Gender**					
Female	37	1	-	1	
Male	30	0.73(0.62-0.85)	<0.001	0.72(0.61-0.85)	<0.001
**Age (year)**					
<45	35.1	1	-	1	-
45-55	33.7	0.94(0.76-1.17)	0.582	0.89(0.71-1.11)	0.289
>55	33.5	0.93(0.74-1.18)	0.546	0.85(0.66-1.09)	0.199
**Educational status (year)**					
≤6	31.7	1	-	1	-
>6	34.7	1.14(0.96-1.36)	0.132	1.12(0.94-1.34)	0.215
**Marital Condition**					
Married	33.9	1	-	1	-
Sig/window	33.7	0.99(0.80-1.22)	0.92	1.04(0.84-1.30)	0.695
**HIV Positive Time (year)**					
<5	25	1	-	1	-
5-10	31.6	1.37(0.69-2.96)	0.38	1.48(0.70-3.29)	0.319
>10	34.4	1.56(0.80-3.26)	0.196	1.37(0.65-3.06)	0.418
**ART Time before second-line (year)**					
<5	33.1	1	-	1	-
5-8	33.3	1.01(0.84-1.21)	0.922	0.93(0.76-1.14)	0.5
>8	35.9	1.13(0.92-1.38)	0.236	1.03(0.80-1.33)	0.791
**Second-line ART Time (year)**					
<3	34.4	1	-	1	-
3-5	33.7	0.97(0.74-1.27)	0.808	0.94(0.69-1.27)	0.672
>5	33.9	0.98(0.76-1.27)	0.869	0.91(0.69-1.22)	0.541
**CM Therapy**					
No	33	1	-	1	-
Yes	37.8	1.24(1.02-1.50)	0.031	1.19(0.97-1.45)	0.087
**CD4 Cell Count (cells/μL)**					
<200	20.7	1	-	1	-
200-350	26.6	1.38(1.00-1.94)	0.051	1.35(0.97-1.89)	0.083
351-500	32.2	1.81(1.33-2.50)	<0.001	1.72(1.26-2.38)	0.001
>500	41.3	2.69(2.01-3.64)	<0.001	2.49(1.85-3.38)	<0.001
**FPG (mmol/L)**					
≤6.2	32	1	-	1	-
>6.2	49	2.04(1.61-2.57)	<0.001	2.08(1.64-2.65)	<0.001
**Hemoglobin (g/L)**					
>110	35.4	1	-	1	-
≤110	24.1	0.58(0.45-0.74)	<0.001	0.59(0.45-0.76)	<0.001

**Abbreviations:** OR, Odds Ratio, CI, Confidence Interval, CM, Chinese Medicine, ART, Antiretroviral Therapy, HIV, Human Immunodeficiency Virus, FPG, Fasting Plasma Glucose.

## Data Availability

Not applicable.

## LIST OF ABBREVIATIONS

PLWHA: Prevalence and Effectors of Hyperlipidemia among HIV/AID Patients
ART: Antiretroviral Therapy
CIs: Confidence Intervals
CVD: Cardiovascular Disease
TG: Elevated Triglycerides
TC: Elevated Total Cholesterol
WHO: World Health Organization
NFATP: National Free Antiretroviral Treatment Program
Hb: Hemoglobin
FPG: Fasting Plasma Glucose
NRTIs: Nucleoside or Nucleotide Reverse Transcriptase Inhibitors
